# Evaluating Mechanisms of Soil Microbiome Suppression of Striga Infection in Sorghum

**DOI:** 10.21769/BioProtoc.5058

**Published:** 2024-09-05

**Authors:** Tamera Taylor, Jiregna Daksa, Mahdere Z. Shimels, Desalegn W. Etalo, Benjamin Thiombiano, Aimee Walmsey, Alexander J. Chen, Harro J. Bouwmeester, Jos M. Raaijmakers, Siobhan M. Brady, Dorota Kawa

**Affiliations:** 1 Department of Plant Biology and Genome Center, University of California, Davis, CA, USA; 2 Plant Biology Graduate Group, University of California, Davis, CA, USA; 3 Netherlands Institute of Ecology (NIOO-KNAW), Department of Microbial Ecology, Wageningen, The Netherlands; 4 Plant Hormone Biology Group, Green Life Sciences Cluster, University of Amsterdam, Amsterdam, The Netherlands; 5 Experimental and Computational Plant Development, Utrecht University, The Netherlands; 6 Plant Stress Resilience, Utrecht University, The Netherlands

**Keywords:** *Striga hermonthica*, Suppressive soils, Microbiome, Sorghum, Aerenchyma, Suberin, Haustorium-inducing factors

## Abstract

The root parasitic weed *Striga hermonthica* has a devastating effect on sorghum and other cereal crops in Sub-Saharan Africa. Available Striga management strategies are rarely sufficient or not widely accessible or affordable. Identification of soil- or plant-associated microorganisms that interfere in the Striga infection cycle holds potential for development of complementary biological control measures. Such inoculants should be preferably based on microbes native to the regions of their application. We developed a method to assess microbiome-based soil suppressiveness to Striga with a minimal amount of field-collected soil. We previously used this method to identify the mechanisms of microbe-mediated suppression of Striga infection and to test individual microbial strains. Here, we present protocols to assess the functional potential of the soil microbiome and individual bacterial taxa that adversely affect Striga parasitism in sorghum via three major known suppression mechanisms. These methods can be further extended to other Striga hosts and other root parasitic weeds.

Key features

• This protocol provides a detailed description of the methods used in Kawa et al. [1].

• This protocol is optimized to assess soil suppressiveness to Striga infection by using natural field-collected soil and the same soil sterilized by gamma-radiation.

• This protocol is optimized to test bacterial (and not fungal) isolates.

• This protocol can be easily extended to other host–parasite–microbiome systems.

## Background

Sorghum (*Sorghum bicolor*) is among the five most important crops in the world and a staple food and forage cereal in Sub-Saharan Africa [2]. Sorghum is grown in diverse agroecological zones, predominantly by small-hold farmers [3]. Most sorghum cultivars are suited to arid, nutrient-depleted soils; yet, sorghum productivity is challenged by pathogens. The parasitic weed *Striga hermonthica* causes substantial sorghum yield losses, affecting approximately 60% of Sub-Saharan African farmlands [4].

Striga is a root parasite that infects cereal crop species, including sorghum, rice, and pearl millet [5]. Striga’s life cycle is dependent on specific compounds exuded from the host roots into the surrounding soil. Striga germinates upon the perception of strigolactones, while haustorium-inducing factors (HIFs) initiate the development of a haustorium, a specialized organ that allows Striga to attach to and penetrate the root tissue [6–8]. Upon reaching the host vasculature, Striga connects its own xylem vessels with its host vasculature [7], enabling the parasite to withdraw water and nutrients from its host, thereby compromising host fitness and productivity [9].

Striga management includes manual weed removal, chemical control methods, and breeding for host resistance [10]. Chemical application poses a challenge in rain-fed agricultural systems, while commercial sorghum varieties show only partial resistance and are not always accessible or suited to the agricultural practices of small-holder farmers [11]. Recently, the functional potential of the soil microbiome to suppress Striga infection has been described. These include microbial isolates that are pathogenic to Striga [12] and those that degrade HIFs or that induce physical barriers to Striga parasitism in host roots [1]. Further development of microbial-based agricultural solutions against Striga will require screening candidate microbes native to soils in Striga-infested regions.

Here, we present a set of protocols to assess the contribution of soil microbiome to Striga infection levels and individual soil-borne bacterial isolates to Striga suppression-associated mechanisms. The soil plug assay enables testing Striga suppressiveness of multiple soils collected from agricultural fields. Striga resistance modes are typically described as pre-attachment resistance [reduced germination and (pre)haustorium formation], and post-attachment resistance (when parasite fails to penetrate the root tissue and/or establish the vascular connection with the host). To distinguish which stage of Striga infection the soil microbiome affects, we recommend extracting and testing host root exudates in an in vitro Striga germination assay and a haustorium formation assay. Preparation of host root cross-sections and their histological staining allows quantification of aerenchyma and suberization, both associated with microbe-mediated suppression of post-attachment stages of Striga infection [1]. To test which isolates affect haustorium formation and induce changes in host root cellular anatomy, we present an in vitro haustorium induction assay and a method of sorghum inoculation in sand, respectively. The presented protocols can be easily adapted and applied to multiple sorghum cultivars and other Striga hosts.

## Materials and reagents

**Biological materials**

1. *Sorghum bicolor* seeds. We recommend using Striga-susceptible sorghum varieties, e.g., Shanqui Red, and the Striga-resistant cultivar SRN39 as positive and negative controls for Striga germination, respectively [13,14] [Shanqui Red: PI 656025, SRN39: PI 656027, available from Germplasm Resource Information Network (GRIN-Global)]
2. *Striga hermonthica* seeds (note that working with Striga in certain locations/countries requires permits (e.g., Federal Noxious Weed Permit issued by APHIS in US) and must be performed in contained-type facilities (e.g., https://crf.ucdavis.edu)
3. Field-collected soil of interest
4. Bacterial isolates of interest

**Reagents**

1. Commercial household bleach [Clorox containing 8.25% (v/v) sodium hypochlorite (NaOCl)]
2. Captan 50% wettable powder (active ingredient: N-Trichloromethylthio-4-cyclohexene-1,2-dicarboximide, 48.9%) (Arysta Life Science, CAS number: 133-06-2)
3. Tween-20 (Sigma-Aldrich, CAS number: 9005-64-5)
4. Calcium nitrate (Sigma-Aldrich, CAS number: 13477-34-4)
5. Potassium nitrate (Spectrum, CAS number: 7757-79-1)
6. Potassium phosphate (Sigma, CAS number: 7778-77-0)
7. Magnesium sulphate (Research Products International, CAS number: 7487-88-9)
8. EDTA (Ethylenediaminetetraacetic acid) ferric salt (Sigma, CAS number: 18154-32-0)
9. Boric acid (Fisher Chemical, CAS number: 10043-35-3)
10. Manganese chloride (J.T. Baker, CAS number: 13446-34-9)
11. Zinc sulphate (Sigma-Aldrich, CAS number: 7446-20-0)
12. Copper sulphate (Spectrum, CAS number: 7758-99-8)
13. Sodium molybdate (Sigma-Aldrich, CAS number: 10102-40-6)
14. Sodium chloride (Fisher Chemical, CAS number: 7647-14-5)
15. Bacto^TM^ yeast extract (Thermo Fisher, catalog number: 212750)
16. N-acetylglucosamine (Sigma-Aldrich, CAS number: 7512-17-6)
17. Tryptic soy broth (TSB), Bacto soybean-casein digest medium (Difco, catalog number: 211825)
18. Bactoagar (agar bacteriological) (Difco, catalog number: 214530)
19. Agarose (VWR, CAS number: 9012-36-6)
20. GR-24rac (StrigoLab, CAS number: 76974-79-3)
21. DMBQ (2,6-methoxy-1,4-benzoquinone), 97% (Sigma-Aldrich, CAS number: 530-55-2)
22. Fluorol yellow 088 (Santa Cruz Biotech., CAS number: 81-37-8)
23. Aniline blue (Fisher Chemical, CAS number: 28631-66-5)
24. Toluidine blue O (J.T. Baker, CAS number: 92-31-9)
25. Lactic acid (Acros Organics, catalog number: 189870010, CAS number: 79-33-4)
26. Syringic acid, 95% (Sigma-Aldrich, CAS number: 530-57-4)
27. Vanillic acid, 98% (Alfa Aesar, CAS number: 121-34-6)
28. Formalin (37% formaldehyde) (Fisher Chemical, CAS number: 50-00-0)
29. 95% ethanol (Koptec, CAS number: 64-17-5)
30. Glacial acetic acid (Fisher Chemical, CAS number: 64-19-7)
31. Glycerol (Fisher Chemical, CAS number: 56-81-5)
32. DMSO (dimethyl sulfoxide) (Sigma-Aldrich, CAS number: 67-68-5)
33. Sodium chloride (Fisher Chemical, CAS number: 7647-14-5)
34. Methanol (Sigma-Aldrich, CAS number: 67-56-1)

**Solutions**

1. Half-strength modified Hoagland media (see Recipes)
2. Bacterial growth media (see Recipes)
3. Formalin-Aceto-Alcohol (FAA) solution (see Recipes)

**Recipes**

1. **Half-strength modified Hoagland media**
  **Table:** 

Stock solution	Reagent	g/L stock	Volume (mL) stock per 1 L of final solution
0.5 M calcium nitrate	Ca(NO_3_)_2_·4H_2_O	118.08	5
1.0 M potassium nitrate	KNO_3_	101.11	2.5
0.1 M potassium phosphate	KH_2_PO_4_	13.609	0.5
0.5 M magnesium sulphate	MgSO_4_·7H_2_O	123.24	2
98.6 mM EDTA ferric salt	C_10_H_12_FeN_2_NaO_8_·3H_2_O	41.52	1
Micronutrient stock:			1
46.3 mM boric acid	H_3_BO_3_	2.86	
9.1 mM manganese chloride	MnCl_2_·4H_2_O	1.81	
0.77 mM zinc sulphate	ZnSO_4_·7H_2_O	0.22	
0.32 mM copper sulphate	CuSo_4_·5H_2_O	0.08	
0.52 mM sodium molybdate	Na_2_MoO_4_·2H_2_O	0.126
  Store at 4 °C.
2. **Bacterial growth media**
  **Table:** 

Reagent	Final concentration	g/L final solution
Sodium chloride	0.5%	5
Potassium dihydrogen phosphate	0.1%	1
Bacto^TM^ yeast extract	0.01%	0.1
N-acetylglucosamine	2 mM	0.44
  Store at room temperature.
3. **Formalin-Aceto-Alcohol (FAA) solution**
  **Table:** 

Reagent	Final concentration	mL/L final solution
Formalin (37% formaldehyde)	10%	100
95% ethanol	50%	500
Glacial acetic acid	5%	50
  Store at room temperature.

**Laboratory supplies**

1. 4 mm mesh
2. 50 mL conical tubes
3. Whatman qualitative filter paper, Grade 1, 90 mm diameter (Sigma-Aldrich)
4. Sterile plastic Petri plate, 100 mm diameter
5. Parafilm
6. Aluminum foil
7. Cones, Depot tree pots 40 cm (Greenhouse Megastore)
8. Gauze pads
9. Rubber bands
10. Ethanol 200 proof (KOPTEC, CAS number: 64-17-5)
11. High-purity filtered sand, effective size 0.45–0.55 mm (Covia)
12. Forceps
13. Pycnometers, 50 mL (KLM BioScientific, Borosilicate 3.3 Glass)
14. Transparent tray
15. Whatman glass microfiber filters, grade GF/A, 13 mm diameter discs (Sigma-Aldrich)

## Equipment

1. Laminar flow cabinet (Labconco, Purifier Biological Safety Cabinet, model: 3440009 LS)
2. Hot stirrer plate (VWR, model: VMS-C7)
3. Stereomicroscope (Nikon, model: SMZ 1500)
4. Balance (Sartorius, Explorer Pro, model: E0114)
5. Orbital shaker (GeneMate, model: Orbital Shaker Variable)
6. Centrifuge for 50 mL tubes (Eppendorf, model: 5810 R)
7. Vibratome (Leica, model: VT1000 S)
8. Fluorescent confocal microscope (Carl Zeiss, model: LSM 700)
9. Vacuum chamber (SP Bel-Art)
10. Microbiological incubator shaker (Innova, model: 4400 Incubator Shaker)
11. Autoclave (Tuttnauer, model 5596SP-1V)
12. Spectrophotometer (Eppendorf, model: BioPhotometer plus)

## Software and datasets

1. ImageJ version 1.53g (https://imagej.net/ij/download.html) with Cell Counter plug-in

## Procedure

- **Sorghum seed surface sterilization and germination**
*Note: Always try to use sorghum seeds that are similar in size.*
  1. Place seeds in a 50 mL conical tube.
  2. Add up to 50 mL of freshly prepared sterilizing solution (30% commercial bleach, 0.2% Tween-20, v/v).
  3. Gently agitate seeds on an orbital shaker in sterilizing solution for 20 min.
  4. Discard the sterilizing solution and seeds that float on the surface of the solution.
  5. Wash seeds with sterile water for 2 min, five times.
  6. Optional: If fungal contamination occurs, agitate seeds overnight in 5% (w/v) Captan slurry followed by five washes in sterile water.
  7. Place seeds on sterile Petri dishes containing two Whatman filter papers moistened with 5 mL of sterile water.
  8. Seal plates with parafilm.
  9. Incubate plates in the dark at 30 °C for the duration indicated per experiment.
- **Striga seed sterilization and preconditioning**
  1. Estimate the density of Striga seeds.
    - Weigh out a small number of dry seeds on a Whatman filter paper in a Petri dish.
    - Record mass.
    - Count the total number of seeds using a stereomicroscope.
    - Calculate the number of seeds per milligram.
    - Repeat steps B1a–d at least three times and calculate an average.
*Note: We recommend conducting this once for each new batch of Striga seeds.*
  2. Wash Striga seeds in 10% (v/v) bleach and 0.02% (v/v) Tween-20 for 10 min.
  3. Discard the solution and wash the seeds five times in sterile water.
  4. Precondition Striga seeds by incubation at 30 °C in the dark. The chemicals and protocols needed for preconditioning are dependent on the experiment and are outlined in their respective sections.
  5. Optional: The number of Striga seeds for each experiment is indicated as the number of germinable seeds. For each newly collected Striga seed batch, germinability should be assessed, as it is highly variable.
  6. Calculate Striga germinability:
    - Sterilize a specific amount (number or weight) of Striga seeds as determined in step B1.
    - Disperse seeds onto a Petri dish with two sterilized Whatman filter papers moistened with 5 mL of sterile water.
    - Precondition at 30 °C in the dark for 10–14 days.
    - Transfer seeds to a new Petri dish with two sterile Whatman filter papers moistened with 5 mL of 1 ppm GR24rac.
    - Seal with parafilm and return to 30 °C in a dark incubator.
    - After three days, count the total number of seeds and number of germinated seeds in the viewing area using a stereomicroscope.
    - Calculate the germination rate for each Petri dish.
      GR% = (Ngs/Nts) × 100
      Ngs: Total number of germinated seeds per Petri dish
      Nts: Total number of seeds per Petri dish
  7. Use these estimates to treat each experiment with the desired amount of germinable Striga seeds using the following formula:
$$Striga\;seeds\;required\;\left(mg\right)=$$
$$\frac{\;\text{number of germinable seeds required per plant }}{\;\text{Striga}\text{ seeds germination percentage }\times \text{ number }\text{Striga}\text{ seeds in 1mg}}\times number\;of\;sorghum\;plants\;to\;be\;infected$$
    - Calculate the total amount of sand needed (you can use 1/10 of required sand amount for preconditioning and mix with the remaining 9/10 on the day of planting).
    - Mix sand with sterile water to reach approximately 16% (v/w) moisture level.
    - Add Striga seeds to wet sand and mix thoroughly.
    - Incubate sand–Striga mix at 30 °C in the dark for 10–14 days.
    - Prepare the sand used as a negative control in the same manner, without adding the Striga seeds.
- **Soil *plug* assay for soil suppressiveness**
**C1. Soil sterilization**
  1. Air-dry the soil at room temperature for 4–7 days.
  2. Sieve the soil through a 4 mm mesh.
  3. Sterilize the soil by gamma irradiation with an 8 kGy dose.
*Notes:*
  - *To attribute the Striga suppressive effect solely to its microbiome, it should be ensured that the physicochemical properties of gamma-sterilized and natural (non-sterilized) soil remain comparable.*
  - *Assess bacterial and fungal diversity to ensure which is depleted.*
**C2. Soil plug preparation**
  1. Mix each soil batch (sterilized and non-sterilized) with sterile water to reach 5% of moisture level (w/v).
  2. Cut a hole at the bottom of a 50 mL conical tube to provide drainage of excess liquid.
  3. Wrap the tubes in aluminum foil or other comparable material to block light. Do not cover the hole at the bottom.
  4. Fill the tubes with soil.
  5. With a 1 mL pipette tip, make a hole in the soil for a germinated sorghum seedling.
  6. Using forceps, gently place a 3-day-old sorghum seedling in the soil.
  7. Place sorghum in the greenhouse or grow in a chamber at 28 °C during the day (11 h) and 25 °C at night (13 h), with a light intensity of 450 μmol/m^2^/s and 70% relative humidity.
  8. Apply 3 mL of sterile deionized water to each soil plug every second day.
  9. Grow sorghum in the soil plug for 10 days.
**C3. Cone preparation**
  1. Sterilize 3,000 germinable Striga seeds per plant as determined in step B5.
  2. Autoclave the cones prior to planting.
  3. Place a gauze pad to cover the hole at the bottom of each cone and secure it with a rubber band.
  4. Fill each cone with 350 mL of fresh (not preconditioned) sand (Figure 1A).
  5. Top up with 350 mL of preconditioned sand without (control) or with Striga seeds.
  6. Within the 50 mL conical cone, make a hole to accommodate the soil plug.
  7. Transfer the 10-day-old sorghum seedling together with the soil plug to the cone (Figure 1A).
  8. Cover the soil plug surface with a thin layer of fresh sand.
  9. Place sorghum in the greenhouse or growth chamber at 28 °C during the day (11 h) and 25 °C at night (13 h) with a light intensity of 450 μmol/m^2^/s and 70% relative humidity.
  10. Apply 50 mL of sterile half-strength modified Hoagland solution to each plant on days 0, 7, and 14. Apply 50 mL of sterile deionized water to each plant on days 1, 4, 10, 13, and 17.
  11. Quantify Striga infection 14 and/or 21 days after transfer to cones.
    **Figure 1.**
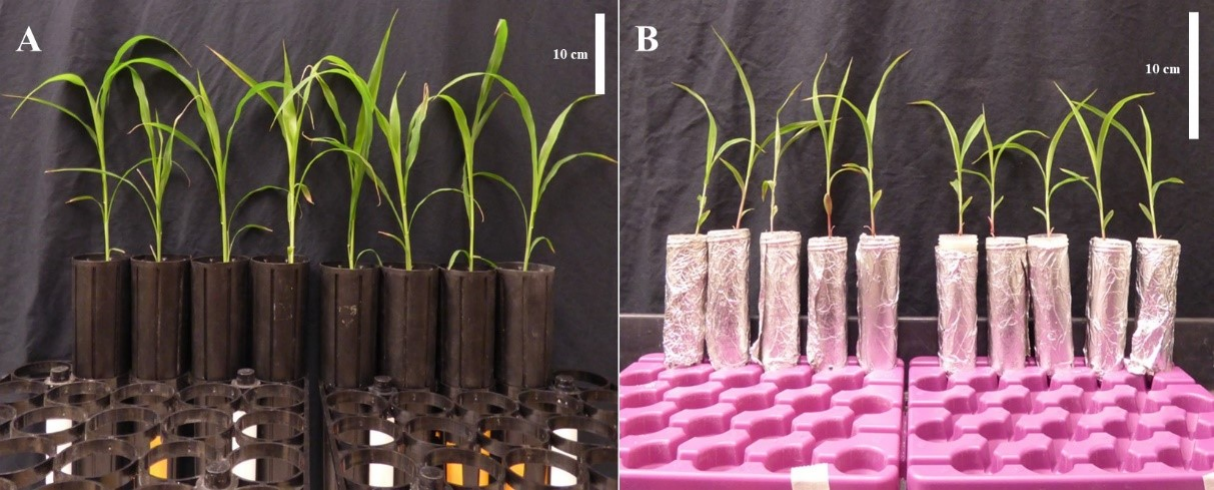
Growth setup to assess soil suppressiveness to Striga in soil plug cone assay (A) and screening for bacterial isolates associated with Striga-suppressive phenotypes (B)
- **Quantification of Striga infection**
  1. Gently remove the plant from the cone.
  2. Shake the plant to remove as much of the sand and soil from the roots as possible. Collect all the soil and sand.
  3. Gently swirl the roots in a tray filled with water to wash off the remaining substrate. Do not rub the roots, since Striga might get de-attached from the host.
  4. Gently dry the roots with a paper towel.
  5. Cut the root system from the shoot.
  6. Record the root’s fresh weight.
  7. Place the root system in a transparent tray filled with water.
  8. Use a stereomicroscope with 10× magnification to examine the roots for sites of Striga attachment and penetration. Use forceps to spread the roots and systematically look for the presence of Striga. Refer to Figures 2D and 2E as to how to distinguish attached from penetrated Striga.
  9. Screen the sand and soil collected in step D2 for any attached Striga that may have fallen off the root, called “de-attached” Striga (see Figure 2C for the developed, penetrated Striga that de-attached from sorghum root).
  10. Sum the number of penetrated Striga on the roots and the number of Striga rescued from the sand (“de-attached Striga”) to obtain the total penetration number.
  11. Sum the number of total penetrated Striga and attached Striga to obtain the total Striga count.
  12. The total Striga count and the total number of attachments should be normalized by the fresh weight of the root scored in step D6.
    **Figure 2.**
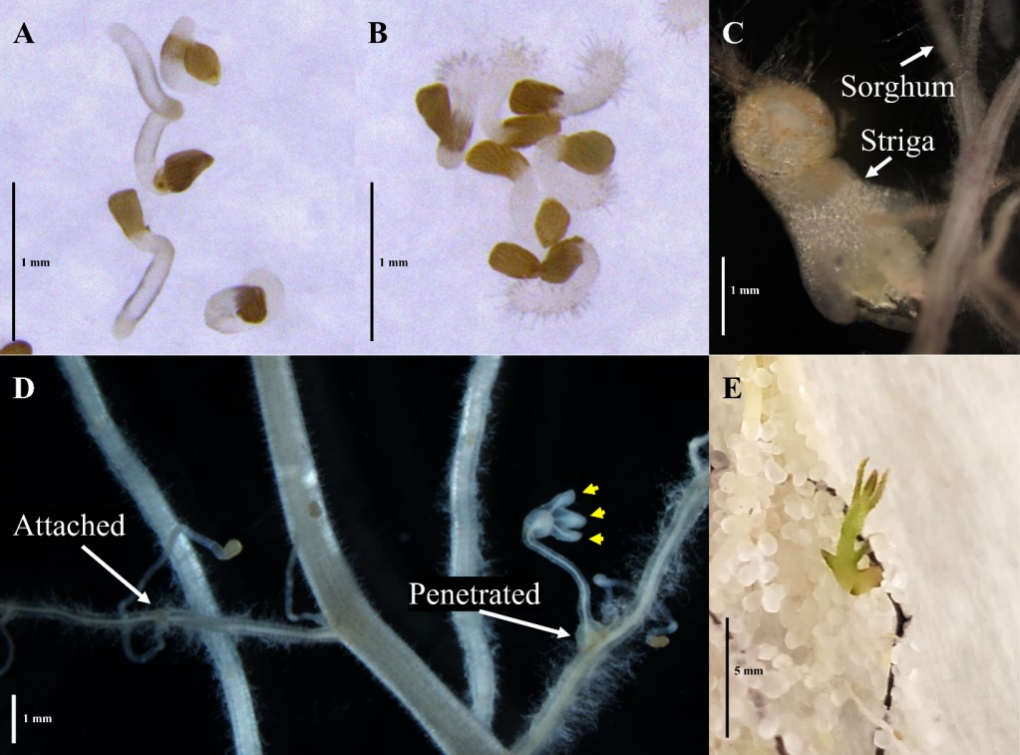
Striga developmental stages of interest. (A) Striga germination and (B) haustorium development of in vitro experiments. (C) Striga may break off at the haustorial connection during harvest even when well-developed. (D) Both attachment and penetrated Striga have developed haustoria that are connected to the sorghum root, but the penetrated Striga shows a further development of leaf lobes. Yellow arrowheads: developing leaf lobes in young, penetrated Striga. (E) Older penetrated Striga may begin developing green leaf tissue.
- **In vitro Striga germination and haustorium formation assay**
**E1. Root exudate collection**
*Note: Exudates are collected from a sorghum plant grown in sterilized soil and a plant grown in non-sterilized soil.*
  1. Place a 1 L beaker under the cone.
  2. Pour 300 mL of sterile water into the cone.
  3. Collect 100 mL of the flowthrough and store refrigerated until use.
**E2. In vitro Striga germination and haustorium formation assay**
  1. Surface sterilize the required amount of Striga seeds as described in section B.
  2. Place a sterile glass fiber filter paper in a 94 mm diameter polystyrene Petri dish and add 3 mL of sterile deionized water.
  3. Spread the sterilized Striga seeds on the Petri dishes with wet filter paper. Use approximately 200 seeds per plate.
  4. Seal the Petri dishes with parafilm.
  5. Precondition Striga seeds for 6–8 days at 30 °C in the dark.
  6. After preconditioning, dry Striga seeds in the Petri dish in the laminar flow hood.
  7. Transfer approximately 30 Striga seeds per well to a 6-well plate.
  8. Add 50–200 times diluted root exudates (as needed to get approximately 40%–50% of Striga seeds to germinate) to the Striga seeds in three technical replicates (exudate collected from an individual plant divided into triplicates) for the germination bioassay. For the haustorium formation bioassay, use undiluted root exudate in triplicates (in the case of the undiluted root exudate, 1 ppm GR24rac is added first to induce uniform germination).
  9. Use 1 ppm GR24rac and 100 mM DMBQ in three technical replicates as a positive control for the Striga seed germination and haustorium formation assays, respectively.
  10. Incubate the 6-well plate in the dark at 30 °C for two days.
  11. Count the number of germinated seeds, seeds that developed haustorium, and the total number of seeds per well. Refer to Figure 2A and 2B for examples of these stages.
  12. Calculate the Striga germination rate (GR):
    GR% = (Ngs/Nts) × 100
    Ngs: Number of germinated seeds per well
    Nts: Total number of seeds per well
  13. Calculate the haustorium formation rate (HFR):
    HFR% = (NHs/Ngs) × 100
    NHs: Total number of haustorium per well
    Ngs: Number of germinated seeds per well
  Striga germination induced by 1 ppm GR24rac is highly dependent on the Striga seed batch, and this germination rate can vary seasonally. In our experiments, 1 ppm GR24rac induces, on average, germination of 60% of Striga seeds.
- **In vitro assay to test the potential of individual bacterial strains to reduce Striga haustorium formation**
**F1. Bacterial inoculum preparation**
  1. Streak the bacterial strain from the glycerol stock onto a plate with a 1/10 strength TSB agar media (1.5% (w/v) agar).
  2. Incubate plates at 26 °C for 24–48 h in the dark.
  3. Prepare liquid bacterial growth media by dissolving the ingredients in Recipe 2 into deionized water.
  4. Autoclave the liquid bacterial growth media for 30 min at 25 °C.
**F2. Striga haustorium formation assay**
  1. Surface-sterilize the required amount of Striga seeds as described in steps B1–B4.
  2. Place two sterile glass fiber filter papers in a 60 mm Petri dish and add 3 mL of sterile deionized water.
  3. Place four 13 mm sterile glass fiber filter papers in each Petri dish.
  4. Add the desired volume of sterile deionized water to surface-sterilized Striga seeds to bring the number of Striga seeds to between 80 and 100 per disc.
  5. Pipette 100 µL of the Striga–water mix prepared in section **E2** to each 13 mm disc.
  6. Seal the Petri dishes with parafilm.
  7. Incubate the plates with the Striga seeds for 11 days at 30 °C in the dark.
  8. At day 12, add 100 µL of water containing a final concentration of 1 µM GR24rac in DMSO to each disc.
  9. Seal the Petri dishes with parafilm and incubate at 28 °C for 48 h.
  10. At day 12, inoculate sterile liquid bacterial growth media with a single colony of the isolate of interest (as described in section **F1**) and incubate for 24 h at 25 °C with shaking at 200 rpm.
  11. Measure OD_600_ using a spectrophotometer.
  12. Centrifuge the liquid culture at 20,000 rcf for 5 min.
  13. Wash the collected cells twice with 10 mL of sterile 0.9% NaCl.
  14. Adjust the OD_600_ to 0.15 by resuspending the cells in 0.9% NaCl.
  15. Transfer 18 μL of the bacterial suspension to 332 μL of the bacterial growth media supplemented with HIFs: 100 µM syringic or 50 µM vanillic acid, dissolved in methanol. Prepare a negative control with the same volume of methanol as used to dissolve the HIFs.
  16. Incubate the culture for another 24 h at 25 °C with shaking at 200 rpm.
  17. Add 50 μL of the cell-free culture filtrate to pre-germinated Striga seeds.
  18. Seal the plates with parafilm and incubate them at 25 °C for 48 h in the dark.
  19. Take pictures of each disc (filter paper with haustoria) under a dissecting microscope.
**F3. Quantification of haustorium formation rate**
  1. Open ImageJ and load the disc (filter paper with haustoria) image file.
  2. Go to *Plugins* and click on *Cell Counter*.
  3. Click on *Initialize*.
  4. Select *Type 1* and add a mark on each non-germinated Striga seed.
  5. Select *Type 2* and add a mark on each germinated Striga seed.
  6. Select *Type 3* and add a mark on each Striga seed that developed haustorium.
  7. Click on *Save Markers*.
  8. Note the counts in the Excel file.
  9. Calculate the haustorium formation rate (HFR) as described in section **E2**.
*Note: The Striga haustorium formation rate should be approximately 70% for 100 µM syringic acid and 30% for 50 µM vanillic acid.*
- **Assay to test the potential of individual bacterial strains to induce root cellular phenotypes associated with Striga suppression**
*Note: Each plant is inoculated with 10^7^ CFU/g sand.*
**G1. Bacterial inoculum preparation**
  1. Streak the bacterial strain from the glycerol stock onto a plate with a 1/10th strength TSB agar media (1.5% w/v agar).
  2. Incubate plates at 26 °C for 24–48 h in the dark.
  3. Inoculate 5 mL of liquid 1/10th TSB with a single colony.
  4. Incubate liquid cultures for 24–48 h at 26 °C with shaking (200 rpm). Culture time depends on the number of cells required.
  5. Calculate the number of cells required per the following formula:
    Number of cells required = 10^7^ (CFU/g soil) × 50 g sand × number of plants to test
  6. Calculate the required volume of the inoculum per the following formula:
    Volume of inoculum required = number of cells required/(CFU/mL of bacterial culture)
  7. Harvest bacteria by centrifugation of the required inoculum volume at 7,000 rcf for 20 min.
  8. Resuspend bacterial pellet in sterile half-strength modified Hoagland solution (5 mL per plant).
**G2. Sorghum inoculation**
  1. Cut a hole at the bottom of the 50 mL conical tube to provide drainage of excess liquid.
  2. Wrap the tubes in aluminum foil. Do not cover the hole at the bottom.
  3. Fill the tubes with sand (approximately 50 g of sand per tube)
  4. Apply 5 mL of sterile half-strength modified Hoagland solution to each tube.
  5. With a 1 mL pipette tip, make a hole for the sorghum seedling.
  6. Pick up a 2-day-old sorghum seedling and, while transferring it to the sand, apply 5 mL of bacterial inoculum (step 8 in section **G1**) directly onto the sorghum root.
  7. Bury the sorghum root and seed with sand.
  8. Place the tube with sorghum in the greenhouse or growth chamber at 28 °C during the day (11 h) and 25 °C at night (13 h) with a light intensity of 450 μmol/m^2^/s and 70% relative humidity.
  9. Apply 5 mL of sterile water every second day.
- **Quantification of root cellular anatomy**
**H1. Root harvesting**
  1. Gently remove a plant from the tube or a cone.
  2. Tap the root to remove the attached sand and soil.
  3. Wash the root system with water.
  4. Cut a 1–1.5 cm segment of the root from the part of interest within the root system.
*Note: We recommend harvesting fragments from the tip and middle section of the crown and seminal root from plants grown in the cone system. For plants grown in 50 mL tubes, harvest individual 1 cm fragments starting 3 cm from the primary root tip in the shootward direction. Avoid root regions that are wavy or damaged. Remove lateral roots from your fragments of interest.*
**H2. Root tissue embedding**
  1. Prepare a 5% agarose gel (m/v with water). We recommend melting the agarose in the autoclave. Keep the agarose stirring on a hot plate to prevent it from solidifying.
  2. Prepare glass vials, each filled with 10 mL of FAA solution.
  3. Pour 5% of the liquid agarose into the Eppendorf tube (Figure 3A, B).
  4. Using forceps, insert the root into the Eppendorf tube containing agarose. Aim to place the root as straight as possible (Figure 3C).
  5. Let the agarose solidify.
  6. Cut off the bottom of the Eppendorf tube with a hot scalpel (Figure 3D).
  7. Open the Eppendorf tube and, with forceps, push the agar “plug” through the hole at the bottom (Figure 3E).
  8. Transfer the agar plug to the glass vial containing FAA (make sure the agar plug is completely covered with solution).
**H3. Root tissue fixation and rehydration**
  1. Place the open glass vial with the submerged agar plug in a vacuum chamber and vacuum infiltrate for 10 min.
  2. Remove the vial from the vacuum chamber, close it, and leave the agar plugs in the FAA solution overnight at room temperature.
  3. Remove FAA from the glass vials.
  4. Add 70% ethanol to the vial (make sure the agar plug is covered in solution) and incubate for 30 min.
  5. Replace 70% ethanol with 50% ethanol and incubate for 30 min.
  6. Replace 50% ethanol with 30% ethanol and incubate for 30 min.
  7. Replace 30% ethanol with 10% ethanol and incubate for 30 min.
  8. Replace 10% ethanol with sterile deionized water.
  9. Store agar plugs in 4 °C.
**H4. Root tissue sectioning**
  1. Cut a 0.5–0.75 cm fragment from the embedded sample with the scalpel, making sure the cut is perpendicular to the root. The root needs to be cut perpendicularly so that it will be 100% vertical when mounted on the specimen disc (Figure 3).
    **Figure 3.**
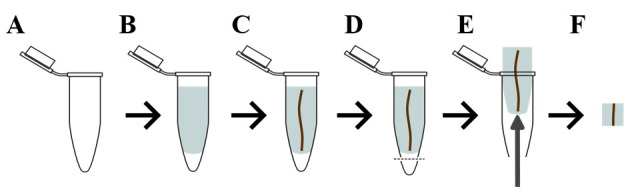
Root embedding process. (A, B) Melted agarose is poured into an Eppendorf tube; (C) then, a section of root is placed into the agarose while still warm. (D) After the agarose solidifies, the bottom is cut off with a hot scalpel, and (E) the agarose plug is pushed out. (F) This plug is cut down further to obtain a straight, perpendicular root portion that is suitable for sectioning on a vibratome.
  2. Mount the agar plug on the specimen disc (provided with the vibratome) with glue (we recommend using Cyanoacrylate glue).
  3. Fill the vibratome buffer tray with ice and pour water over it.
  4. Attach the specimen disk with the sample to the buffer tray.
  5. Place the knife securely into the knife holder and attach it to the vibratome. We recommend wearing cut-proof gloves during this step.
  6. Set the required knife amplitude and speed. We recommend starting with amplitude 8 and speed 8 and adjusting it depending on the tissue structure.
  7. Set the section thickness to 300 μm. If your tissue keeps popping out from the agar, increase the section thickness.
  8. Make the first section and discard it.
  9. Make the next section and capture it gently with the fine-tipped paintbrush.
  10. Observe the section under a 10× magnification stereomicroscope. Discard sections that are blurry or damaged.
  11. Place the section in the 12-well plate filled with water and store it on ice.
  12. Prepare 3–4 sections per sample for each staining type.
**H5. Suberin quantification**
  1. Suberin staining and image acquisition
    - Replace the water in the 12-well plate with 0.01% Fluorol yellow (in lactic acid, w/v).
    - Incubate at room temperature for 30 min in the dark with gentle agitation.
    - Remove the Fluorol yellow solution.
    - Wash sections for 5 min with deionized water. Repeat three times.
    - Add 0.5% aniline blue solution (in water, w/v).
    - Incubate at room temperature for 30 min with gentle agitation.
    - Wash sections for 10 min with deionized water. Repeat four times.
    - Mount sections on microscopy slides with 50% glycerol (v/v).
    - Image sections with a confocal laser scanning microscope with an excitation wavelength of 488 nm. The gain should be adjusted manually. (Image sections where you expect the highest signal at first to set the gain.)
    - Save file as a raw image file (.czi file in case of Zeiss 700).
  2. Suberin content quantification
    - Open ImageJ and load root cross-section raw image file.
    - Use the *Freehand selections* tool to outline endodermal cells with representative fluorescent signals.
    - Use the *Measure* tool under *Analyze* to record the *Mean Gray Value*.
    - Repeat steps H5.2b–c for 3–4 representative cells.
    - Average the *Mean Gray Value* for quantified cells to express mean fluorescent signal.
  3. Developmental patterns of suberin quantification
*Note: To assess the influence of microbes on the developmental status of suberization, one needs to first assess the distance from the root tip where the patchy and fully suberized zone starts for a given growth condition and genotype.*
    - Open the cross-section image in the image processing software.
    - Mark the regions where the signal is weaker due to the technical challenges of obtaining sorghum root sections that can be visualized in a single plane. These regions can be recognized by following the changes in the background fluorescent signal from the vasculature. Exclude the regions with less fluorescence in the vasculature and adjacent endodermal cells.
    - Count the number of suberized cells (excluding the regions marked in the above step) (Figure 4A, B).
    - Count the total number of endodermal cells.
    - Calculate the proportion of suberized cells in the endodermis.
    - Calculate the proportion of plants with a fully suberized endodermis.
**H6. Aerenchyma quantification from sections**
*Note: Fluorol yellow sections can be used in the brightfield microscope to quantify aerenchyma. If using the same sections as for suberin quantification, proceed to step H6.1e.*
  1. Image acquisition
    - Replace the water in the well plate with sections with 0.1% toluidine blue (m/v in deionized water).
    - Incubate at room temperature for 5 min with gentle agitation.
    - Remove the toluidine blue solution.
    - Wash the sections for 1 min with deionized water. Repeat five times.
    - Image sections with a brightfield microscope and save the image in .tiff format.
  2. Aerenchyma quantification
    - Open ImageJ and load root cross-section .tiff file.
    - Use the *Freehand selections* tool to outline the aerenchyma (Figure 4C).
    - Use the *Measure* tool under *Analyze* to record the area of each aerenchyma lacuna outlined.
    - Use the *Add Noise* tool under *Process* to mark already recorded areas.
    - Use the *Freehand selections* tool to outline and record the total root cross-section area (TRA).
    - Save the *Results* report as a .csv file with a name that indicates the image analyzed.
    - Calculate the total aerenchyma area (TAA) as a sum of individual aerenchyma lacunae quantified in step 2c.
    - Calculate aerenchyma proportion per formula:
      Aerenchyma proportion = TAA/TRA
    We typically observe aerenchyma formed in 30% of the root area (for crown roots of 4-week-old sorghum grown in a soil-plug system).
    **Figure 4.**
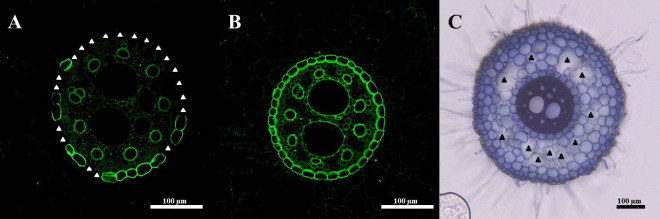
Example quantification images. (A) The endodermis may be partially or (B) fully suberized. (C) Aerenchyma lacuna outlines are traced to measure total aerenchyma content. White triangles: not suberized endodermal cells; black triangles: aerenchyma lacuna.
  3. Aerenchyma quantification—porosity assay for plants grown in 50 mL tubes
    - Fill in pycnometers with water and weigh them (P_w_).
    - Gently remove the plant from the tube.
    - Gently tap the root system to remove sand.
    - Wash the root system in water, trying to remove as much sand as possible without squeezing the root.
    - Very gently dry the root system with a paper towel.
    - Weigh the root system (R).
    - Transfer the root system to the pycnometer, refill the pycnometer with water, and weigh (P_r_).
    - Place the open pycnometer with the root system in the vacuum chamber and vacuum infiltrate until the last air bubbles can be seen. A longer time is required for larger root systems. Shake the pycnometers a few times during infiltration to aid in the release of air bubbles.
    - Weigh the pycnometer with the root and water after vacuum infiltration (P_v_).
    - Calculate the porosity per the following formula:
      Root system porosity = (P_v_ - P_r_)/ (P_w_ + R - P_r_)
  An average root porosity of 8.4% in mock (sterile media) treatment is typically observed for the entire root system of 10-day-old sorghum plant.

## Validation of protocol

This protocol or parts of it has been used and validated in the following research article(s):

- Kawa et al. [1]. The soil microbiome modulates the sorghum root metabolome and cellular traits with a concomitant reduction of Striga infection. Cell Reports. (Figure 1, panel C–E; Figure 3, panel B–G, Figure 4 panel A–H)

## References

[1] Kawa D., Thiombiano B., Shimels M. Z., Taylor T., Walmsley A., Vahldick H. E., Rybka D., Leite M. F., Musa Z., Bucksch A., .(2024). The soil microbiome modulates the sorghum root metabolome and cellular traits with a concomitant reduction of Striga infection. Cell Rep. 43(4): 113971. 38537644 10.1016/j.celrep.2024.113971 PMC11063626

[2] Paterson A. H., Bowers J. E., Bruggmann R., Dubchak I., Grimwood J., Gundlach H., Haberer G., Hellsten U., Mitros T., Poliakov A., .(2009). The Sorghum bicolor genome and the diversification of grasses. Nature. 457(7229): 551-556. 19189423 10.1038/nature07723

[3] Spallek T., Mutuku M. and Shirasu K. (2013). The genus Striga: a witch profile. Mol Plant Pathol. 14(9): 861-869. 23841683 10.1111/mpp.12058 PMC6638688

[4] Ejeta G. and Gressel J. (2007). Integrating New Technologies for Striga Control. World Scientific. ISBN: 978-981-270- 708-6.

[5] Runo S. and Kuria E. K. (2018). Habits of a highly successful cereal killer, Striga. PLoS Pathog. 14(1): e1006731. 29324906 10.1371/journal.ppat.1006731 PMC5764402

[6] Cui S., Wada S., Tobimatsu Y., Takeda Y., Saucet S. B., Takano T., Umezawa T., Shirasu K. and Yoshida S. (2018). Host lignin composition affects haustorium induction in the parasitic plants *Phtheirospermum japonicum* and *Striga hermonthica* . New Phytol. 218(2): 710-723. 29498051 10.1111/nph.15033

[7] Yoshida S., Cui S., Ichihashi Y. and Shirasu K. (2016). The Haustorium, a Specialized Invasive Organ in Parasitic Plants. Annu Rev Plant Biol. 67(1): 643-667. 27128469 10.1146/annurev-arplant-043015-111702

[8] Bouwmeester H., Li C., Thiombiano B., Rahimi M. and Dong L. (2021). Adaptation of the parasitic plant lifecycle: germination is controlled by essential host signaling molecules. Plant Physiol. 185(4): 1292-1308. 33793901 10.1093/plphys/kiaa066 PMC8133609

[9] Graves J. D., Press M. C. and Stewart G. R. (2006). A carbon balance model of the sorghum‐*Striga hermonthica* host‐parasite association. Plant Cell Environ 12(1): 101-107.

[10] Goldwasser Y. and Rodenburg J. (2013). Integrated Agronomic Management of Parasitic Weed Seed Banks. In: Joel, D. M., Gressel, J. and Musselman, L. J.(Eds.). *Parasitic Orobanchaceae*. Springer Berlin Heidelberg. 393–413.

[11] Jamil M., Kountche B. A. and Al-Babili S. (2021). Current progress in *Striga* management. Plant Physiol. 185(4): 1339-1352. 33793943 10.1093/plphys/kiab040 PMC8133620

[12] Nzioki H. S., Oyosi F., Morris C. E., Kaya E., Pilgeram A. L., Baker C. S. and Sands D. C. (2016). Striga Biocontrol on a Toothpick: A Readily Deployable and Inexpensive Method for Smallholder Farmers. Front Plant Sci. 7: e01121. 10.3389/fpls.2016.01121 PMC4976096 27551284

[13] Gobena D., Shimels M., Rich P. J., Ruyter-Spira C., Bouwmeester H., Kanuganti S., Mengiste T. and Ejeta G. (2017). Mutation in sorghum *LOW GERMINATION STIMULANT 1* alters strigolactones and causes *Striga* resistance. Proc Natl Acad Sci USA. 114(17): 4471-4476. 28396420 10.1073/pnas.1618965114 PMC5410831

[14] Kawa D., Taylor T., Thiombiano B., Musa Z., Vahldick H. E., Walmsley A., Bucksch A., Bouwmeester H. and Brady S. M. (2021). Characterization of growth and development of sorghum genotypes with differential susceptibility to *Striga hermonthica* . J Exp Bot. 72(22): 7970-7983. 34410382 10.1093/jxb/erab380 PMC8643648

